# The Acute Effect of Multi-Ingredient Antioxidant Supplementation following Ionizing Radiation

**DOI:** 10.3390/nu15010207

**Published:** 2023-01-01

**Authors:** Donald Xhuti, Irena A. Rebalka, Mahek Minhas, Linda May, Kieran Murphy, Joshua P. Nederveen, Mark A. Tarnopolsky

**Affiliations:** 1 Department of Pediatrics, McMaster University Health Sciences Centre, Hamilton, ON L8N 3Z5, Canada; 2 Exerkine Corporation, McMaster University Medical Centre (MUMC), Hamilton, ON L8N 3Z5, Canada; 3 Department of Pathology and Molecular Medicine, McMaster University, Hamilton, ON L8S 4L8, Canada; 4 Department of Kinesiology, McMaster University, Hamilton, ON L8S 4L8, Canada; 5 Department of Medical Imaging, University of Toronto, Toronto, ON M5S 2C5, Canada; 6 Cora Therapeutics, Toronto, ON M5K 1N2, Canada

**Keywords:** antioxidants, ionizing radiation, γ-H2AX, mtDNA

## Abstract

Radiation exposure is an undeniable health threat encountered in various occupations and procedures. High energy waves in ionizing radiation cause DNA damage and induce reactive oxygen species (ROS) production, which further exacerbate DNA, protein, and lipid damage, increasing risk of mutations. Although endogenous antioxidants such as superoxide dismutase have evolved to upregulate and neutralize ROS, exogenous dietary antioxidants also have the potential to combat ionizing radiation (IR)-induced ROS production. We evaluated a cocktail of ingredients (AOX) purported to have antioxidant and mitochondrial protective properties on the acute effects of IR. We show that IR stimulates DNA damage through phosphorylation of DNA repair proteins in the heart, brain, and liver of mice. AOX showed partial protection in brain and liver, through a lack of significant activation in given repair proteins. In addition, AOX attenuated the IR-induced increase in NF-kβ mRNA and protein expression in brain and liver. Lastly, cytochrome *c* oxidase complex transcripts were significantly higher in heart and brain following radiation, which was also diminished by prior ingestion of AOX. Together, our findings suggest that a multi-ingredient AOX supplement may attenuate the IR-induced cellular damage response and represents a feasible and cost-effective preventative supplement for at-risk populations of radiation exposure.

## 1. Introduction

Radiation exposure is a concern for many populations and professions, as high energy waves, particularly ionizing radiation (IR), alter chemical structure in cells [1], induce reactive oxygen species (ROS) [2], and are involved in tissue damage [3] and increased risk of cancer [4]. Specifically, due to their persistent exposure to IR, nuclear industry workers, aircrews, medical radiology technicians, and patients of frequent diagnostic imaging may be at increased risk [5,6]. Indeed, it has been estimated that in some developed countries, over 3% of cancers can be attributed to radiation exposure from diagnostic imaging [7]. In addition, with recent concerns about nuclear accidents rising due to miliary conflict, there is a risk of excessive radiation exposure to the local populations, potentially affecting long term health [8,9].

The mutagenic effects and cellular damage associated with IR are primarily caused by two cellular events [10,11,12]. First, radiation induces double stranded breaks in DNA, which cause mutations, loss of heterozygosity, and can lead to cancer or cell death. Imperative to repair the breaks, a damage repair response is triggered where proteins are phosphorylated and localized in the nucleus to initiate the repair. These proteins include γ-H2AX and ataxia telengiectasia mutated (ATM), and together with cell cycle arrest proteins, cyclin-dependent kinase inhibitor 1A (CDKN1A) and tumor protein 53 (TP53), have been proposed as the top four biomarker candidates of IR-induced DNA damage [12]. In addition to nuclear DNA damage, IR can also cause damage to mitochondrial DNA (mtDNA), which may be more detrimental to the health of the cell, as mtDNA lacks the excision and recombination repair mechanism [13], making it more susceptible to mutagenesis [14].

Secondly, IR-induced effects include the generation of ROS, which oxidize molecules and cause further damage to DNA, lipids, and proteins in the cell [10]. The high energy waves from IR interact with water molecules in the cell generating H_2_O^+^ and a free electron, which subsequently produces free radicals and ROS [15]. Molecular damage induced by ROS will then further exacerbate DNA damage and initiate an inflammatory response [16]. As protection against ROS, cells have evolved to endogenously produce free scavenger proteins such as superoxide dismutase (SOD), which break down and neutralize ROS, making SOD an endogenous antioxidant [17]. Exogenous free scavengers can also be supplemented with dietary antioxidants and have been shown to be protective against IR-induced DNA damage [18,19]. Previous studies in patients undergoing diagnostic imaging have shown that ingesting oral antioxidants prior to their procedure can prevent nuclear DNA damage in blood cells, as reflected in an attenuation of γ-H2AX [19,20]. However, whether similar protection can be conferred to mitochondrial DNA has yet to be investigated. In our current study, we created a formula containing 10 different ingredients with antioxidant properties, which have been shown to be beneficial against radiation-induced damage by reducing lethality [21] or attenuating markers of DNA and cellular damage [18,19,20], including quercetin [22,23], astaxanthin [24], zeaxanthin [25], vitamin C [26], vitamin B_12_ [27], selenium [28], folate [29], CoQ10 [30,31], α-lipoic acid [32], and vitamin E [33]. Importantly, we specifically chose to include CoQ10, vitamin E, and α-lipoic acid, as they were the main components of a multi-ingredient supplement that we have shown lowered lactate and oxidative stress markers in patients with primary mitochondrial myopathy [34]. Consequently, the novelty of our proposal is that we have proposed a multi-ingredient supplement that would target multiple final common pathways of IR-induced damage including mitochondrial DNA and membrane protection. We also chose to evaluate brain and heart given that these have been shown to be negatively affected by repeated doses [35,36] and even a single [37] dose of IR exposure.

Thus, the aim of our study was to test the hypothesis that our multi-ingredient antioxidant supplement (AOX) would provide cellular protection to rodents exposed to a single high dose of IR (2 Gy). We employed varying molecular biology techniques to assess nuclear and mitochondrial DNA damage, inflammatory response, and mitochondrial stress response, following acute high dose IR in mice. We provide evidence that partial cellular protection may be achieved when ingesting AOX prior to IR exposure. This AOX represents a feasible, cost-effective therapeutic intervention that has the potential to reduce the increased risk in mutagenesis when exposed to IR. We specifically designed the study to interrogate the potential for the supplement to prophylactically protect against IR exposure that would be of relevance to patients undergoing diagnostic imaging, radiation workers and those at risk of nuclear accidents.

## 2. Materials and Methods

Animals and study design. All animal experiments and procedures were performed with the approval of McMaster University Animal Research Ethics Board and conformed to the standards of the Canadian Council on Animal Care (AUP#20-04-17). Male C57BL/6J mice were ordered from Jackson Laboratories (Bar Harbor, ME, USA) and randomly assigned to one of two separate cohorts, acute (+30 min) or later (+24 h) post-IR collection times. Furthermore, each of these two groups were further divided into one of three study groups: CON (no RAD nor AOX), RAD, or RAD + AOX. Animals were maintained in standard mouse cages (3 mice per cage) on an *ad libitum* diet of standard chow throughout the experimental period. Mice were fasted for 12 h prior to gavage feeding and experimental procedures. An antioxidant cocktail (based around the provision of nuclear and mitochondrial DNA protection, Table 1), was administered to RAD + AOX mice via oral gavage two hours prior to irradiation. The RAD group received a vehicle control of corn oil and water. The control group (CON) was not gavage fed. Animals in the RAD and RAD + AOX groups were also subjected to 2 Gy of whole-body radiation (Gammacell^®^ 3000, Best Theratronics, Kanata, ON, Canada). Animals in the acute and later cohorts were sacrificed at +30 min and +24 h post radiation exposure, respectively. The blood, liver, heart, and brain were rapidly removed, snap-frozen, and stored at −80 °C until analysis. Samples intended for flow cytometry were incubated in 1 mL of buffer containing 10 g/L collagenase B (Roche, Basel, Switzerland, 11088831001), 4 g/L Dispase II (Roche, 494207800) and Ham’s F-10 media, at 37 °C.

*Flow cytometry*. Flow cytometry technique was performed on liver, heart, and brain cells, as well as bone marrow (BMCs) and peripheral blood mononuclear cells (PBMCs). Prior to fixation, cells were stained with LIVE/DEAD^®^ Fixable Dead Cell Stain Kit (ThermoFisher Scientific, Waltham, MA, USA) viability dye for 10 min at 4 °C in the dark. Samples were then washed and fixed with BD Cytofix/Cytoperm Buffer™ (BD Bioscience, Mississauga, ON, Canada, 554714) to permeabilize the cells, incubating at 4 °C for 20 min. After fixation, cells were washed, centrifuged at 500× *g* for 5 min and cell pellets were resuspended in 100 μL BD Perm/Wash™ buffer (BD Bioscience, 554714). Cells were incubated with Phospho-Histone H2A.X (Ser139) (20E3, Cell Signaling Technology, Danvers, MA, USA) Rabbit mAb (Alexa Fluor^®^ 488 Conjugate) antibody (Cell Signaling Technology, 9719S) for 1 h at 4 °C in the dark. Following incubation, cells were washed and resuspended in 400 µL PBS (ThermoFisher Scientific, 14190250) and spun at 500× *g* for 5 min at 4 °C. Tissues with a prominent pellet were resuspended in up to 300 μL PBS while PBMCs and BMCs were resuspended in 150 μL PBS. The cell suspension was filtered through a 100 µm cell strainer prior to analysis. All samples were immediately analyzed using a flow cytometer (MoFlo™ XDP, Beckman Coulter, Brea, CA, USA).

*Immunoblotting.* Approximately 20 mg of snap-frozen tissue was homogenized in Axygen™ Snaplock Microcentrifuge tubes (14-222-156, Fisher Scientific, Waltham, MA, USA) via the FastPrep-24 Tissue and Cell Homogenizer in chilled RIPA lysis and extraction buffer (ThermoFisher Scientific, 89901) supplemented with protease and phosphatase inhibitor cocktail (Halt™, ThermoFisher Scientific, 78440). The homogenate was centrifuged for 20 min at 4 °C and 12,000× *g*, followed by removal of the supernatant. Total protein concentrations were determined using a BCA Protein Assay Kit (Pierce, Thermo Scientific, 23225) and protein samples were prepared in 1× Laemmli Buffer (Fisher Scientific, AAJ61337AC). Prior to gel electrophoresis, samples were heated at 95 °C for 5 min and micro-centrifuged. Immunoblots were resolved using a 4–20% Criterion TGX Precast Midi Protein Gel (5671094, Bio-Rad Laboratories, Hercules, CA, USA) with a PageRuler Plus Prestained Protein Ladder at 10 to 250 kDA (ThermoFisher Scientific, 26619). All blots were run in 1× Tris/Glycine/SDS buffer (Bio-Rad Laboratories, 1610772). Protein samples were transferred to a membrane using Trans-Blot Turbo Midi Nitrocellulose Transfer Packs (Bio-Rad Laboratories, 1704159EDU) and subsequently stained with Ponceau S solution (P7170-1L, Sigma, St. Louis, MO, USA) for 5 min. Membranes were imaged using the ChemiDoc MP Imaging System (Bio-Rad Laboratories) and placed in blocking solution (5% BSA in 1× TBS) for 1 h. Blots were left to incubate in primary antibody at 4 °C overnight with constant agitation. All primary antibodies were prepared in 5% BSA in TBS (Table 2). Membranes were serially washed (3 × 5 min) in 1× TBST and incubated with the appropriate secondary antibody at 1:20,000 (diluted in 5% BSA in 1× TBS) for 1 h. Membranes were serially washed again (3 × 5 min) in 1× TBST, then incubated with Clarity Max Western ECL substrate (Bio-Rad Laboratories, 1705062) for 5 min at room temperature (RT). All membranes were visualized using the ChemiDoc MP Imaging System (Bio-Rad Laboratories). Furthermore, band intensities were quantified using Image Lab 6.1 software (Bio-Rad Laboratories) and normalized to Ponceau S staining.

*RNA isolation*. RNA was isolated from 20 mg of tissue using the Trizol/RNeasy method. All samples were homogenized with 500 μL Trizol Reagent with the FastPrep-24 Tissue and Cell Homogenizer (MP Biomedicals, Solon, OH, USA) for 40 s at a speed of 6 m/s. All samples were incubated at room temperature for 5 min. 10µL chloroform was added to each sample, mixed vigorously for 30 s, incubated at RT for 3 min, and spun at 12,000× *g* for 15 min at 4 °C. The aqueous phase containing RNA was transferred to a new tube, precipitated with 250 µL isopropanol, incubated for 10 min at 4 °C, and centrifuged at 12,000× *g* for 10 min at 4 °C. The pellet was immediately resuspended in 500 µL 75% ethanol, vortexed briefly, and centrifuged at 7500× *g* for 5 min at 4 °C. Prior to solubilization, the pellet was air dried for 10 min. The pellet was resuspended in 30 µL RNase-free water. RNA concentration (ng/mL) and purity (260/280) were determined with the Nano-Drop 1000 Spectrophotometer (ThermoFisher Scientific).

*cDNA Synthesis.* Reverse transcription was performed on 1 μg cell lysate of RNA using a SuperScript™ IV VILO™ Master Mix (ThermoFisher Scientific, 11756500), as per the manufacturer’s protocol. The reaction mixtures were incubated in a T100 Thermal Cycler (Bio-Rad Laboratories) with thermocycling conditions of 25 °C for 10 min (Annealing), 50 °C for 10 min (Elongation), and 85 °C for 5 min (Enzyme Inactivation). Synthesized cDNA was diluted with nuclease-free water to obtain a concentration of 5.5 ng/µL prior to storage at −80 °C.

*Quantitative PCR.* Gene expression was determined by running 4.5 µL of cDNA template (5.5 ng/µL) to a final volume of 10 uL on a CFX384 Real-Time System (Bio-Rad Laboratories) using the TaqMan Fast Advanced Master Mix (ThermoFisher Scientific, 4444557), using the following TaqMan cycling conditions: 20 s at 95 °C for initial denaturation, followed by 40 cycles with 3 s denaturation at 95 °C, and 30 s annealing and elongation at 60 °C. The fluorescence threshold was automatically set above the background level. Fold-increase in mRNA probes of interest (Table 3) was calculated by the 2^−ΔΔCt^ method and expression levels were normalized to the reference gene *B2M* (*Beta-2-microglobulin*). The absence of genomic contamination was ensured using an NTC control for each probe. All RT-qPCR reactions were run in duplicates.

*LORD-Q assay*. Tissue fragments from heart, brain, and liver were homogenized in lysis buffer using the FastPrep-24 Tissue and Cell Homogenizer (MP Biomedicals). DNA was then isolated and purified, as per manufacturer instructions with DNeasy Blood and Tissue Kit (69506, Qiagen, Hilden, Germany). DNA concentration (ng/mL) and purity (260/280) was determined by spectrometric analysis using the Nano-Drop 1000 Spectrophotometer (Thermo Scientific). Samples were then diluted to 7.5 ng/uL where 1 µL was used per 10 µL reaction. In addition, the rt-PCR reaction contained forward and reverse primers for mtDNA, 1× KAPA2G Fast Hot Start ReadyMix, and LightCycler 480 ResoLight dye. The ResoLight dye is provided at 20× concentration, which was optimized and diluted to 0.2×. Primers used for amplification of the mitochondrial short (S) and long (L) DNA fragment are listed in Table 4. mtDNA lesions were derived and calculated from C_t_ (C_p_) scores of short (C_pS_) and long fragments (C_pL_), using the formula below (1). Non-irradiated mice were used as the control (*Ref*). Amplification efficiencies (E) for both primer sets were determined through a standard curve with serial dilutions of 24, 16, 8, 4, 2, 1, 0.5 and 0.25 ng/uL. This protocol was adapted from Lehle et al. (2014), and Dannenmann et al. (2017) [38,39].

(1) $$\frac{\mathrm{Lesions}}{10\;\mathrm{kb}}=\left[{\left(\frac{\frac{E_{L}{}^{Cp_{L}}}{{}^{E_{S}{}^{Cp_{S}}}}}{{\left(\frac{E_{L}{}^{Cp_{L}}}{{}^{E_{S}{}^{Cp_{S}}}}\right)}_{Ref}}\right)}^{\left(\frac{1}{length_{L}}\right)}-1\right]\times 10,000$$

## 3. Results

### 3.1. Ionizing Radiation Causes Acute DNA Damage across Multiple Tissues 30 Min following Exposure

**Figure 1:**
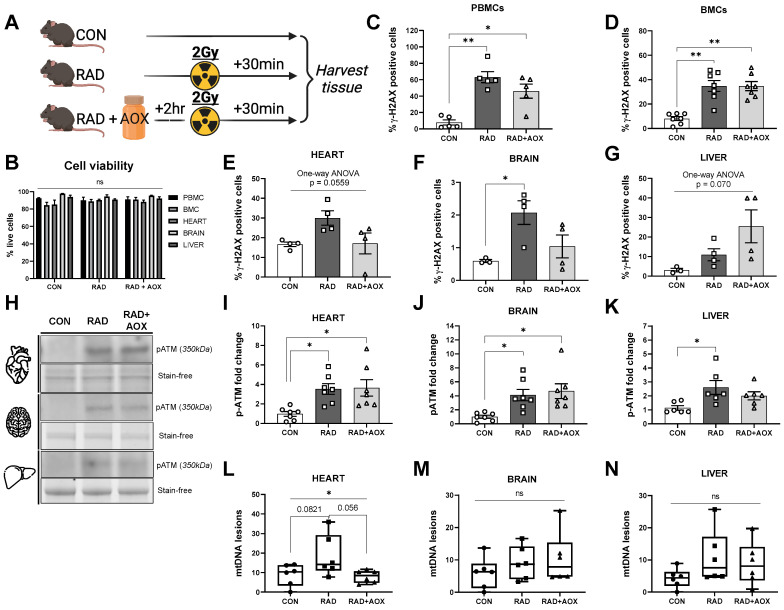
Ionizing radiation causes DNA damage across multiple tissues in mice. (**A**) Design of experiment. Mice were harvested 30 min following 2 Gy radiation. 2 h prior, mice were gavage fed with vehicle placebo (RAD) or antioxidant (RAD + AOX). Control mice (CON) were not gavage fed or irradiated. Flow cytometry of (**B**) viable cells, stained for γ-H2AX were analyzed and represented as % of positive cells in (**C**) Peripheral blood mononuclear cells (PBMCs), (**D**) Bone marrow cells (BMCs) (**E**) heart, (**F**) brain, and (**G**) liver. (**H**) Representative Western blot images for p-ATM protein expression and densitometric quantification in the (**I**) heart, (**J**) brain and (**K**) liver. Protein expression data is expressed as fold change mean (±SEM) relative to CON. (**L**–**N**) mtDNA lesions were analyzed and graphed through LORD-Q assay. Individual data points shown. Statistical analysis was done with One-way ANOVA with Tukey’s post hoc. *, ** denote a significant difference between groups of *p* < 0.05, and *p* < 0.01, respectively.

A higher percentage of γ-H2AX-positive cells was observed, as a result of IR, across multiple tissues (Figure 1C–G). In isolated PBMCs, the percentage of γ-H2AX cells significantly increased from 7.9% (CON) to 63.1% in RAD, while a significant increase to 46.2% was observed in RAD + AOX animals (Figure 1C). Similarly, BMCs isolated from mouse femurs saw a significant increase in γ-H2AX-positive cells following radiation, in both RAD (34.8%) and RAD + AOX (34.7%) groups, compared to CON (8.1%) (Figure 1D). In the heart, brain, and liver, the percentage of γ-H2AX positive cells was higher in RAD and RAD + AOX groups than in CON, however, only in brain tissue did this increase reach statistical significance (Figure 1E–G). Evidently, the employed dose of IR was sufficient to induce a cellular response detectable by the utilized outcome measures.

Higher pATM expression was found in RAD and RAD + AOX groups compared to CON across all three interrogated tissues (Figure 1H–K). The fold-changes of pATM in RAD mice relative to CON for heart, brain, and liver were 3.6, 4.1, and 2.5-fold, respectively (*p* < 0.05). pATM expression was also higher in RAD + AOX animals compared to CON heart and brain tissue. Specifically, pATM expression was 3.6-fold higher in the heart (*p* < 0.05), and 4.6-fold in the brain (*p* < 0.05); whereas, in liver, there was no significant difference in pATM levels between RAD + AOX and CON.

Using a previously validated method [39] of long-run real-time PCR (LORD-Q) mtDNA damage quantification, we found a significant main effect between groups in heart tissue, where RAD was trending towards significantly higher mtDNA lesions compared to CON (*p* = 0.082) (Figure 1L). Importantly, heart tissue of RAD + AOX mice did not show a higher number of mtDNA lesions vs. CON (Figure 1L). No significant effect of IR was observed upon mtDNA lesions in the brain and liver of either RAD or RAD + AOX mice compared to CON (Figure 1M,N).

### 3.2. Multi-Ingredient Antioxidant Supplement Dampens the Inflammatory Stress Response Induced 24 h following Ionizing Radiation

To investigate the protective effect of AOX upon IR-induced DNA damage, we measured the mRNA abundance of inflammatory transcription factors *NF-κβ*, *STAT3*, and *CDKN1A* in heart, brain, and liver harvested 24 h following IR.

**Figure 2:**
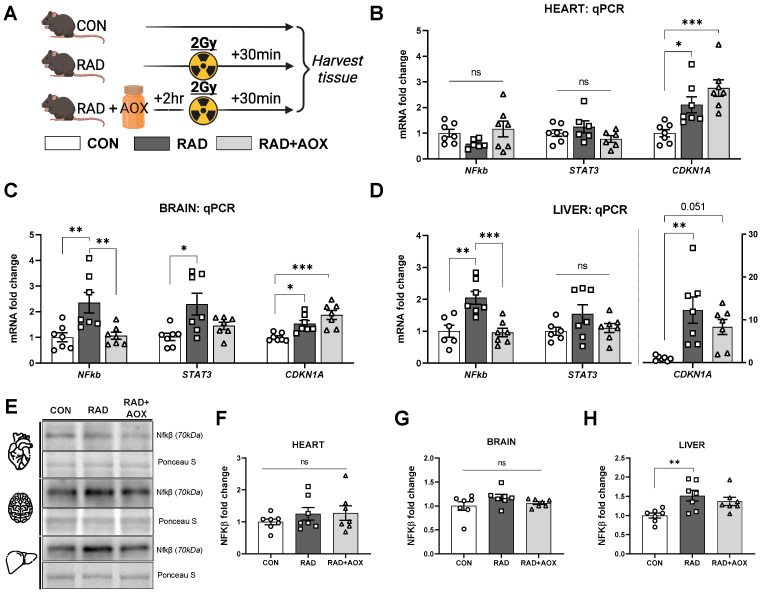
Antioxidant multi-ingredient supplement attenuates increase in NF-kβ induced by ionizing radiation. (**A**) Design of experiment. Mice were harvested 24 h following 2 Gy radiation. 2 h prior to IR exposure, mice were gavage with vehicle placebo (RAD) or antioxidant (RAD + AOX). Control mice (CON) were not gavage fed or irradiated. The mRNA abundance of; *NF-kβ*, *STAT3*, and *CDKN1A* in the (**B**) heart, (**C**) brain, and (**D**) liver were measured using qRT-PCR. Analysis was done using the 2^−Δ^ method normalized to *B2M* housekeeper gene. (**E**) Representative Western blot images for NFkβ protein expression and densitometric quantification in the (**F**) heart, (**G**) brain and (**H**) liver. Data is expressed as fold change mean (±SEM) relative to CON. Individual data points shown, *n* = 7 per group. Statistical analysis was done with One-way ANOVA with Tukey’s post hoc. *, **, and *** denote a significant difference between groups of *p* < 0.05, *p* < 0.01, and *p* < 0.001, respectively.

Relative to CON, NF-κβ mRNA abundance was significantly higher in RAD animals in both the brain and liver by 2.3 and 2.0-fold, respectively. RAD + AOX animals showed no significant difference compared to CON (Figure 2B–D). Relative to CON, significantly higher (1.51-fold-change) NF-κβ protein content was observed in the RAD group, with RAD + AOX showing no significant difference (Figure 2H). In contrast, NF-κβ protein content was not significantly different across the three groups in heart or brain (Figure 2F,G).

The mRNA abundance of *STAT3* transcripts were significantly higher in brain tissue of RAD mice (2.3-fold), with no significant elevation in the RAD + AOX group. Heart and liver tissues did not show a significant difference in *STAT3* transcription between groups (Figure 2B–D).

The cell cycle arrest transcription factor *CDKN1A* was higher in RAD than CON in all tissues (2.1, 1.6, and 12.2-fold-change for heart, brain, and liver, respectively; Figure 2B–D). In contrast to results for other transcripts, the IR-induced increase in *CDKN1A* mRNA transcripts was not attenuated in RAD + AOX (Figure 2B–D).

### 3.3. Multi-Ingredient Antioxidant Supplement Attenuates Increase in Mitochondrial and Nuclear Encoded Mitochondrial Transcripts 24 h following Radiation Induced Damage

**Figure 3:**
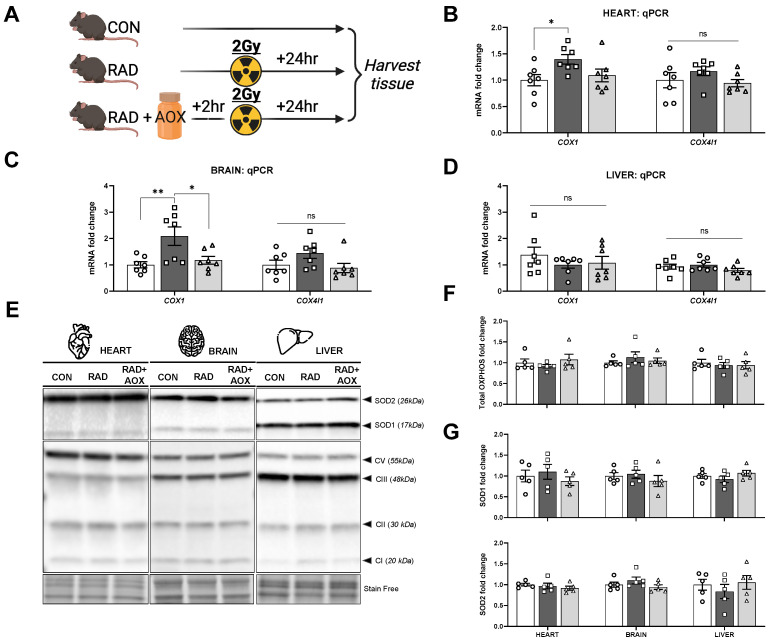
Antioxidant multi-ingredient supplement attenuates increase in *COX* mRNA abundance. (**A**) Design of experiment. Mice were harvested 24 h following 2 Gy radiation. 2 h prior, mice were gavaged with vehicle placebo (RAD) or antioxidant (RAD + AOX). Control mice (CON) were not gavage fed or irradiated. mRNA abundance for genes of mitochondrial complex proteins I and IV in the (**B**) heart, (**C**) brain, and (**D**) liver were measured using qRT-PCR. Analysis was done using the 2^−Δ^ method normalized to *B2M* housekeeper gene. (**E**) Representative Western blot images for superoxide dismutase I/II proteins (SODI/II) and total OXPHOS proteins, and (**F**,**G**) densitometric quantification in the heart, brain, and liver. Data is expressed as fold change mean (±SEM) relative to CON. Individual data points shown. Statistical analysis was done with One-way ANOVA with Tukey’s post hoc. *, and ** denote a significant difference between groups of *p* < 0.05, and *p* < 0.01, respectively.

The mRNA abundance of the mtDNA encoded *COX1* subunit was higher in RAD vs. CON for both heart (1.4-fold) and brain (2.1-fold), with no radiation-induced elevations observed in RAD + AOX animals vs. CON (Figure 3B,C). Expression of the nuclear DNA encoded transcript (*COX4I1*), showed a similar pattern for both heart and brain, but did not reach statistical significance. No effect of IR was found on mRNA abundance for either *COX1* or *COX4I1* in the liver (Figure 3D). Given the mRNA abundance changes, total protein content for representative mitochondrial electron transport proteins were evaluated, however no differences between groups were observed in any of the tissues tested (Figure 3F). Additionally, no IR-effect was observed on total protein content of endogenous antioxidant proteins SODI/II 24 h after IR exposure, in heart, brain, or liver (Figure 3G).

## 4. Discussion

The current study provides evidence for the potential benefit of an AOX multi-ingredient supplement to attenuate some of the cellular consequences of IR exposure, mainly in more terminally differentiated tissues, but also in peripheral blood mononuclear cells.

Specifically, we showed that IR induced DNA damage in isolated PBMCs, BMCs, heart, brain, and liver cells via staining of γ-H2AX, which was partially attenuated with AOX. Furthermore, ingestion of AOX attenuated the IR-induced increase in NF-kβ transcript and protein, as well as mitochondrial protein complex transcripts. The dose of IR administered in the present study (2 Gy) is higher than the typical dose of exposure during nuclear medicine procedures (~0.02 Gy for PET/CT scan); however, it is known that prolonged and repeated IR exposure can nevertheless compound within the body and lead to adverse effects in the heart and brain [23,40]. Survivors of radiation therapy display elevated prevalence of cardiac fibrosis and increased risk of coronary artery disease, cardiomyopathy, arrhythmia, and other cardiovascular complications [40]. Cognitive and memory dysfunction are also prevalent in patients undergoing radiation therapy, which may accelerate age-related dementia [41]. Given these consequences of radiation, a protective antioxidant supplement could be of potential benefits to cosmic radiation (X-ray, particle-based radiation) and other frequent radiation exposures encountered during occupational or diagnostic events. In contrast, radiation doses at or above 2 Gy are possible following nuclear disasters such as nuclear reactor accidents or nuclear war exposure [9,42,43]. Consequently, prophylaxis with the current formulation could potentially attenuate some of the cellular consequences of such high exposures.

Indeed, earlier studies of patients undergoing diagnostic imaging have shown promising results. Velauthapillai and colleagues showed that when taking oral antioxidants prior to technetium-99m methylene diphosphonate (^99m^Tc MDP) bone scans, an attenuation of the increase in percentage of γ-H2AX foci was observed in isolated PBMCs when compared to PBMCs isolated from individuals that did not preemptively supplement with antioxidants, suggesting that antioxidants taken prior to radiation exposure may prevent DNA injury [19]. In addition, when incubating human blood lymphocytes with a cocktail of antioxidants before administering a CT-scan-comparable dose of radiation (0.02 Gy), antioxidant-treated cells show a lower percentage of γ-H2AX positive foci, again, suggesting a protection of antioxidant supplementation [18]. Literature highlighting individual antioxidant ingredients within our AOX formula further supports the idea of partial nuclear DNA protection against IR. In particular, blood lymphocytes incubated in various antioxidants for 1 h prior to IR, namely selenium, vitamin E, vitamin C, and CoQ10, have all demonstrated significant 12–25% reductions in γ-H2A foci number compared to placebo treated cells [20]. However, perhaps the most convincing evidence supporting the assertion that antioxidants may provide protection against radiation-induced DNA damage comes from randomized controlled trials of antioxidant supplementation studies in cancer patients undergoing radiation therapy [44]. Studies supplementing with vitamin E (ingredient used in our study) have reported a significant 38% reduction in radiation-induced adverse effects [45]. However, counterproductively, they also report reductions in local tumor control rates, and even more concerning, patients receiving antioxidants had poorer overall survival, suggesting that antioxidants do not only provide protection for healthy cells, but also cancer cells, thereby reducing the effectiveness of radiation therapy [45]. Although meta-analyses have concluded that antioxidants may not be a feasible supplement during radiation therapy, their use may indeed provide protection to tissue when given prior to IR exposure [45], in situations such as diagnostic imaging, nuclear accidents, or in workers exposed to radiation (i.e., interventional radiologists, flight crews, etc.).

In addition to protecting against nuclear DNA damage, protection to mitochondrial DNA damage is crucial due to limited repair mechanisms of mtDNA and its increased rate of mutagenesis [13,14]. Indeed, a potent stress signal such as 2 Gy of IR may cause an increase in cytosolic Ca^2+^ [46,47], which can activate mitochondrial phosphatases. This in turn dephosphorylates the cytochrome *c* protein complex thereby removing the inhibition on ATP production, increasing respiration and mitochondrial membrane potential to elevate ROS production. Excess ROS may then cause further damage to mtDNA and nuclear DNA. We saw elevations in mtDNA lesions in the heart of RAD mice compared to CON and RAD + AOX mice. In addition, *COX1* and *COX4I1* mRNA abundance were robustly (*COX1*) and trending (*COX4I1*) higher in heart and brain tissue of RAD animals, but not in those treated with AOX and exposed to IR when compared to CON. Numerous studies have shown induction of mtDNA lesions by radiation, both ionizing and ultraviolet [39,48], likely causing a mitochondrial-stress response accompanied by an increase in transcription of COX complex protein transcripts. Given the mitochondrial-related consequences of radiation, our antioxidant formula contains ingredients (CoQ10, vitamin E, and α-lipoic acid) that have been shown to lower oxidative stress and improve mitochondrial function in mitochondrial myopathy patients [34]. Interestingly, within the current study, AOX attenuated the increase in mtDNA lesions in heart and *COX* mRNA transcripts in heart and brain. In liver tissue, however, no changes in gene transcripts were detected following radiation. The liver possesses greater regenerative properties, with increased rate of proliferation [49,50], possibly accounting for observed discrepancies in the 24 h timepoint following radiation. Indeed, we chose to assess our endpoints immediately post, and 24 h post radiation for two reasons; namely, to assess immediate protein activity (phosphorylation of H2AX and ATM), and to allow for response in gene transcription, 24 h post radiation. However, given the different rates of cellular turnover in different tissues [50], the 24 h timepoint could have been too late to see differences in liver *COX* mRNA transcripts, whereas an investigation 12 h post radiation may have allowed for the observation of significant changes. In addition, although not significant, our results suggest a trend towards an increase in mtDNA lesions in the liver immediately after radiation. Interrogating an intermediary timepoint, possibly 12 h post radiation, may have allowed more time for mtDNA lesions to develop and be observed. Together, we provide evidence that our formula may be beneficial and protective against mitochondrial-related radiation-induced damage, in heart, brain and possibly liver given a more inclusive timeline.

Furthermore, it stands to reason that combining several individual antioxidant supplements may have a synergistic effect, not only related to nuclear and mitochondrial DNA protection, but also other radiation-mediated consequences such as inflammation, and possibly even greater benefits with continuous administration of AOX. The inflammatory response is a protective mechanism by which the immune system responds to foreign pathogens and damaged cells [51]. Herein, gene expression of inflammatory transcription factor *NF-kβ* was significantly higher in liver and brain of RAD mice 24 h post-IR. This translated into an upregulation in protein expression in liver specifically. It is well known that *NF-kβ* is activated following radiation [52]. It has been shown that IR activates NF-kB, which in turn is involved in promoting invasion and migration of cancer cells, in vitro [53]. Inhibitors of the transcription factor have been proposed as targets to minimize and reduce the adverse effects of radiation mediated by NF-kβ [54]. Interestingly, in the current study, our AOX ameliorated the increase in mRNA abundance and protein expression following radiation in brain and liver tissue. Although it is difficult to discern the specific mechanism, ingredients within our AOX have been shown in the literature to inhibit NF-kβ. In particular, quercetin inhibits osteoclastic differentiation and potentially protects against bone loss via inhibition of an NF-kβ-dependent pathway [55]. In addition, CoQ10 has also been shown to successfully ameliorate increases in NF-kβ and IL-6 in intestinal tissue of irradiated mice [30].

Collectively, our study provides evidence that a single dose of a multi-ingredient antioxidant supplement can have some beneficial effects in vivo, by partially negating the consequences of radiation exposure. Although not tested in our current investigation, it is possible that multiple intermittent doses of antioxidants prior to exposure may result in greater protection. In addition, the timing of oral gavage feeding represents a challenge, as it is possible that different ingredients have different timing required to be bioavailable to tissue. Furthermore, studies of continuous administration of antioxidants following high exposure to radiation increased survival in mice [21,56], suggesting that supplementation following exposure may further increase the beneficial effects of AOX observed within the current investigation. Future studies may investigate the effects of continuous AOX administration, pre- and post-IR, in mice or humans to further elucidate the beneficial effects of these combined ingredients. Nevertheless, the collective results presented here suggest that oral antioxidants can offer a feasible option to attenuate the cellular damage incurred by radiation exposure when given prophylactically. Populations at high risk of accidental radiation exposure may benefit from a prophylactic supplementation strategy.

## 5. Patents

PCT/CA2020/051439 filed at the European patent and trademark office (PCT) covers the formulation used in this research work.

## Figures and Tables

**Table 1:** Multi-ingredient antioxidant supplement list.

Ingredient	Dose (mg/kg)	Vendor	Cat. Number
Quercetin	41	Cayman Chemical Company	10005169
CoQ10	27.5	MyBioSource LLC	MBS165643
α-Lipoic Acid	27.5	Sigma-Aldrich	T1395
Vitamin E	27.5	Sigma-Aldrich	T3126
Vitamin C	41	Sigma-Aldrich	A5960
Astaxanthin	0.82	Sigma-Aldrich	SML0982
Zeaxanthin	0.51	Cayman Chemical Company	10009992
Folate	0.082	Sigma-Aldrich	F7876
Selenium	0.0205	Sigma-Aldrich	229865-5G
Vitamin B_12_	0.0103	Sigma-Aldrich	V6629-250MG

**Table 2:** Antibody information.

**Primary Antibodies**				
**Antibody**	**Species**	**Vendor**	**Cat. Number**	**Dilution**
pATM	M	Thermo Scientific	MA1-2020	1:3000 in 5% BSA in 1× TBS
γH2AX	R	CST	9718S	1:5000 in 5% BSA in 1× TBS
NF-kβ	R	CST	4764T	1:1000 in 5% BSA in 1× TBS
SODI/II	R	abcam	ab16831	1:1000 in 5% BSA in 1× TBS
Total OXPHOS	M	abcam	ab110411	1:1000 in 5% BSA in 1× TBS
**Secondary Antibodies**				
**Antibody**		**Vendor**	**Cat. Number**	**Dilution**
Peroxidase AffiniPure Donkey Anti-Mouse IgG (H + L)		Jackson ImmunoResearch	715-035-151	1:20,000 in 5% BSA in 1× TBS
Peroxidase AffiniPure Donkey Anti-Rabbit IgG (H + L)		Jackson ImmunoResearch	711-035-152	1:20,000 in 5% BSA in 1× TBS

**Table 3:** TaqMan probe gene assay ID.

Gene Symbol	Gene Name	Vendor	Assay ID
*B2M*	Beta-2-microglobulin	Thermo Fisher	Mm00437762_m1
*NF-kβ2*	Nuclear factor of kappa light polypeptide gene enhancer in B cells 2	Thermo Fisher	Mm00479807_m1
*CDKN1A*	Cyclin-dependent kinase inhibitor 1A (P21)	Thermo Fisher	Mm00432448_m1
*STAT3*	Signal transducer and activator of transcription 3	Thermo Fisher	Mm01219775_m1
*COX1*	Cytochrome c oxidase subunit 1	Thermo Fisher	Mm04225243_g1
*COX4I1*	Cytochrome c oxidase subunit 4I1	Thermo Fisher	Mm01250094_m1

**Table 4:** LORD-Q primers.

Locus	Base Pairs	Primer Denotation	Primer Sequence
mtDNA (L) mouse	3921	MM.mtDNA.F	5′-TCCTACTGGTCCGATTCCAC-3′
		MM.mtDNA.L.R	5′-CGGTCTATGGAGGTTTGCAT-3′
mtDNA (S) mouse	74	MM.mtDNA.F	5′-TCCTACTGGTCCGATTCCAC-3′
		MM.mtDNA.S.R	5′-GGCTCCGAGGCAAAGTATAG-3′
*Col1a1* (L) murine	2637	MM.col1a1.L1.F	5′-CCGTTTGTCCCATTACTGCT-3′
		MM.col1a1.L1.R	5′-AGCAAGGACGAGGACTTTGA-3′
*Col1a1* (S) murine	60	MM.col1a1.S.F	5′-AAAGTGGGAATCTGGACACG-3′
		MM.col1a1.S.R	5′-CAGAGGCCTTATTTCATTTTCG-3′

## Data Availability

Data can be made available from corresponding author upon reasonable request.
